# Development and validation of nomograms to intraoperatively predict metastatic patterns in regional lymph nodes in patients diagnosed with esophageal cancer

**DOI:** 10.1186/s12885-020-07738-9

**Published:** 2021-01-05

**Authors:** Fei Zhao, Rong-Xin Lu, Jin-Yuan Liu, Jun Fan, Hao-Ran Lin, Xiao-Yu Yang, Shu-Hui You, Qian-Ge Wu, Xue-Yun Qin, Yi Liu, Fu-Xi Zhen, Jin-Hua Luo, Wei Wang

**Affiliations:** Department of Thoracic Surgery, First Affiliated Hospital of Nanjing Medical University, 300 Guangzhou Road, Nanjing, 210029 China

**Keywords:** Esophageal cancer, Regional lymph node, Metastasis, Multivariable logistic model, Nomogram

## Abstract

**Background:**

An accurate intraoperative prediction of lymph node metastatic risk can help surgeons in choosing precise surgical procedures. We aimed to develop and validate nomograms to intraoperatively predict patterns of regional lymph node (LN) metastasis in patients with esophageal cancer.

**Methods:**

The prediction model was developed in a training cohort consisting of 487 patients diagnosed with esophageal cancer who underwent esophagectomy with complete LN dissection from January 2016 to December 2016. Univariate and multivariable logistic regression were used to identify independent risk factors that were incorporated into a prediction model and used to construct a nomogram. Contrast-enhanced computed tomography reported LN status and was an important comparative factor of clinical usefulness in a validation cohort. Nomogram performance was assessed in terms of calibration, discrimination, and clinical usefulness. An independent validation cohort comprised 206 consecutive patients from January 2017 to December 2017.

**Results:**

Univariate analysis and multivariable logistic regression revealed three independent predictors of metastatic regional LNs, three independent predictors of continuous regional LNs, and two independent predictors of skipping regional LNs. Independent predictors were used to build three individualized prediction nomograms. The models showed good calibration and discrimination, with area under the curve (AUC) values of 0.737, 0.738, and 0.707. Application of the nomogram in the validation cohort yielded good calibration and discrimination, with AUC values of 0.728, 0.668, and 0.657. Decision curve analysis demonstrated that the three nomograms were clinically useful in the validation cohort.

**Conclusion:**

This study presents three nomograms that incorporate clinicopathologic factors, which can be used to facilitate the intraoperative prediction of metastatic regional LN patterns in patients with esophageal cancer.

**Supplementary Information:**

The online version contains supplementary material available at 10.1186/s12885-020-07738-9.

## Background

Esophageal cancer is the eighth most common cancer and the sixth leading cause of cancer-related death worldwide [1]. It is important for surgeons to determine the accurate patterns of lymph node metastasis in patients with esophageal cancer because lymph node metastasis will affect patient prognosis and decide appropriate treatment strategies [2, 3]. However, it is controversial for surgeons to choose the best strategy for lymph node dissection.

Generally, surgeons commonly use contrast-enhanced computed tomography (CT) as a preoperative work-up to determine esophageal cancer staging. With technological developments, positron emission tomography CT (PET-CT) and endoscopic ultrasound (EUS) are regarded as additional methods to improve the diagnostic accuracy in cases of lymph node metastasis. Unfortunately, some researchers find and demonstrate lack of reliability in PET-CT and EUS staging [4, 5]. The diagnostic accuracy of PET-CT in the context of regional lymph nodal metastasis is controversial because of the relatively low sensitivity of the technique. Furthermore, in patients with tuberculosis, false-positive lymph nodes in esophageal cancer will always be found because the specificity of nodal staging may be reduced [6, 7]. Meanwhile, additional diagnostic methods such as EUS cannot be routinely used to screen patients and are only performed when surgeons are informed by radiologists of their suspicion of metastatic lymph nodes by contrast-enhanced CT [8, 9].

Although surgeons prefer to choose the extended systematic nodal dissection as the best way to provide accurate pathological nodal staging and remove all possible metastasis of lymph nodes, the possibility of omitting positive lymph nodes also exists and the occurrence of postoperative complications increases. There are two opposite opinions about extended systematic nodal dissection. The benefit of extended systematic nodal dissection is under intense discussion [10, 11]. Surgeons aim to identify an accurate strategy of lymph node dissection to improve prognosis and reduce the possibility of postoperative complications in patients with esophageal cancer.

Regional lymph node maps for esophageal cancer were revised in the 8th edition of tumor–node–metastasis (TNM) staging [12]; lymph nodes were classified into three regions: cervical, thoracic, and abdominal. For increased accuracy, we divided the thoracic region into three regions: the upper thoracic region: 2R/2 L and 4R/4 L; the middle thoracic region: 7, 8 U, and 8 M; and the lower thoracic region: 8Lo,9R/9 L, and 15. Few studies have been performed to explore different patterns of regional lymph nodes. If strategy of lymph node dissection can be designed by obtaining accurate patterns of regional lymph nodes metastasis, patients would benefit substantially.

However, there are different clinical and pathological characteristics in each patient intraoperatively diagnosed with esophageal cancer. Many researchers have demonstrated that individual clinical parameters and the histological components of the tumor will greatly influence the occurrence of lymph node metastasis [13–15]. Metastatic patterns of regional lymph nodes will be also affected by patient heterogeneity.

Therefore, the aim of the present study was to identify clinicopathologic characteristics and to develop and validate three nomograms that incorporated these risk factors to intraoperatively predict patterns of regional lymph nodes in patients with esophageal cancer. These nomograms will provide surgeons with additional guidance to make appropriate decisions for lymph node dissection and minimize damage to patients.

## Methods

### Patient selection

In our medical center, 760 patients underwent operations for esophageal cancer in 2016, and of these, we excluded 73 patients from this study because of low-quality medical records. Of the remaining 687 patients, 200 were excluded because they did not meet the study inclusion criteria. The remaining 487 patients were enrolled in this retrospective analysis as an independent training cohort. Patients were intraoperatively diagnosed with esophageal cancer by rapid frozen-section pathological analysis and diagnosis. We used the 8th edition of the TNM staging for esophageal cancer to define the pathological stage of all patients [16]. In 2017, 596 patients with esophageal cancer underwent operations at our medical center, and of these, we excluded 96 patients from this study because of low-quality medical records. Of the remaining 500 patients, 294 were excluded by using the same criteria as used for the training cohort. An independent validation cohort of 206 consecutive patients was constructed. Patients with any one of the following characteristics were excluded: 1) distant metastasis by contrast-enhanced CT and further confirmed by PET-CT; 2) receiving chemotherapy or radiotherapy preoperatively; 3) diagnosing as tuberculosis preoperatively; 4) the number of stations dissected in operation did not meet the current standards of complete lymph node dissection (i.e., all lymph node stations, including 1R, 2R, 4R, 7, 8 U, 8 M, 8Lo, 9R, 15, 16, 17, 18, 19, and 20 in the right side and 1 L, 2 L, 4 L, 7, 8 U, 8 M, 8Lo, 9 L, 15, 16, 17, 18, 19, and 20 in the left side); 5) other types of cancer.

Contrast-enhanced chest CT and whole abdominal CT, brain magnetic resonance imaging, and bone scintigraphy were used as routine preoperative assessment for patients. We used contrast-enhanced CT to determine preoperative N-staging. All patients received three-incision esophagectomy as the approach for esophageal cancer resection.

Tissue specimens comprised esophagus-containing tumors and lymph nodes. Esophageal cancers were analyzed by sending rapid frozen sections to the Pathology Department at our medical center. The diagnostic criteria in rapid frozen sections are similar to that in permanent sections. Lymph-vascular space invasion is defined as the invasion of cancer to the lymphatic and vascular spaces or in the lumens. Neural invasion is defined as the invasion of cancer to the space surrounding or in the peripheral neural plexus. We used 10% formalin to fix remaining tumor and lymph nodes, and pathological department performed conventional formalin-fixed, paraffin-embedded pathological tests postoperatively.

### Statistical analysis

SPSS v23 (IBM Corp.) was used for statistical testing. Continuous variables with abnormal distributions were presented as medians (range). We used the Mann–Whitney U test to compare groups of data. Categorical data is described as the count (percentage) and we used chi-squared or Fisher’s exact test to compare difference. In the training cohort, univariate analysis was used to evaluate the significance of associations with the three patterns of regional lymph node metastases (*P* < 0.20). Independent predictors were determined by further analyzing these significant variables by multivariable logistic analysis (*P* < 0.05). we calculated Odds ratios (ORs) with 95% confidence intervals (CIs). We used the rms package in R v3.5.1 (http://www.r-project.org/) to generate three nomograms of multivariable analysis results. The area under the curve (AUC) of the receiver operating characteristics (ROC) curve was used to quantify the nomogram prediction accuracy and the null hypothesis is AUC = 0.5. Calibration curves was used to assess the Consistency between actual patient outcomes and predicted outcomes. In the validation cohort, the AUC and calibration curves were performed using the same methods. We used the rmda package in R to depict Decision curve analysis (DCA) based on the net benefit. A two-tailed *P* value of < 0.05 was considered statistically significant.

## Results

### Basic clinicopathologic characteristics

In this study, a total of 693 patients diagnosed with esophageal cancer was enrolled and were separated by different years into a training cohort (*n* = 487) in 2016 and a validation cohort (*n* = 206) in 2017. The ratio of patients in the training and validation cohorts was almost 2:1. The clinicopathologic characteristics of individuals in the training and validation cohorts are outlined in Table 1. There were no significant differences in the clinical characteristics between the training and validation cohorts (*P* > 0.05). Lymph node metastasis positivity affirmed by routine pathology was 43.1% (210/487) in the training cohort and 35.9% (74/206) in the validation cohort, respectively. The validation cohort is justified as a better population of patients to verify the practicability of the nomogram. The CT report of lymph node status provided in the validation cohort was confirmed by two radiologists at our center. Lymph node metastasis positivity suspected by contrast-enhanced CT was only 8.25% (17/206), which is less than that affirmed by routine pathology (35.9%; 74/206).

**Table 1:** Clinicopathologic characteristics

	Number (percentage)/Median (range)			
	Total patients	Training cohort	Validation cohort	
Variables	(***n*** = 693)	(n = 487)	(n = 206)	***P*** value
Age(years)	64 (41–84)	64 (42–84)	64 (41–82)	0.908
Sex (Female/Male)	164 (23.7%)/529 (76.3%)	125 (25.7%)/362 (74.3%)	39 (18.9%)/167 (81.1%)	0.063
Smoke (1:Yes/ 0:No)	319 (46.3%)/370 (53.7%)	237 (48.7%)/250 (51.3%)	82 (23.7%)/120 (76.3%) Missing 4 (1%)	0.054
Alcohol (1:Yes/ 0:No)	256 (37.2%)/432 (62.8%)	192 (39.4%)/295 (60.6%)	64 (31.8%)/137 (68.2%) Missing 5 (2%)	0.069
Gastritis/Esophagitis (1:Yes/ 0:No)	22 (3.2%)/664 (96.8%)	13 (2.7%)/474 (97.3%)	9 (4.5%)/190 (95.5%) Missing 7 (3%)	0.140
Diabetes (1:Yes/ 0:No)	41 (6%)/647 (94%)	27 (5.5%)/460 (94.5%)	14 (7%)/187 (93%) Missing 5 (2%)	0.481
Tumor History (1:Yes/ 0:No)	17 (2.5%)/671 (97.5%)	13 (2.7%)/474 (97.3%)	14 (2%)/187 (98%) Missing 5 (2%)	0.789
Family History Of Tumor (1:Yes/ 0:No)	75 (10.9%)/613 (89.1%)	60 (12.3%)/427 (87.7%)	15 (7.5%)/186 (92.5%) Missing 5 (2%)	0.080
length of Tumor (cm)	3.36 (3.17–3.56)	3.37 (3.13–3.59)	3.36 (2.97–3.76)	0.996
Blood Type (A, B, AB, O)	214 (31.8%)/269 (40%) /0 (0%)/190 (28.2%)	138 (29.1%)/200 (42.1%) /0 (0%)/137 (28.8%) Missing 12 (2%)	76 (38.4%)/69 (34.8%)/ 0 (0%)/53 (26.8%) Missing 8 (4%)	0.077
Neutrophil(G) (1:> 3.3/0:< 3.3)10^9^/L	349 (50.8%)/338 (49.2%)	238 (49.5%)/243 (50.5%) Missing 6 (1%)	111 (53.9%)/95 (46.1%)	0.318
Lymphocyte(G) (1:> 1.6/0:< 1.6)10^9^/L	349 (50.8%)/338 (49.2%)	239 (49.5%)/242 (50.3%) Missing 6 (1%)	110 (53.4%)/96 (46.6%)	0.405
N/L(G) (1:> 1.5/0:< 1.5)	343 (50%)/343 (50%)	238 (49.6%)/242 (50.4%) Missing 7 (1%)	105 (51%)/101 (49%)	0.830
Platelet(G) (1:> 187/0:< 187)10^9^/L	330 (48%)/357 (52%)	234 (48.6%)/247 (51.4%) Missing 6 (1%)	96 (46.6%)/110 (53.4%)	0.677
N/P(G) (1:> 0.02/0:< 0.02)	350 (50.5%)/343 (49.5%)	242 (49.7%)/245 (50.3%)	108 (52.4%)/98 (47.6%)	0.561
ALP(G) (1:> 82/0:< 82)U/L	351 (51.2%)/334 (48.8%)	237 (49.5%)/242 (50.5%) Missing 8 (2%)	114 (55.3%)/92 (44.7%)	0.182
APTT(G) (1:> 26.6/0:< 26.6) S	347 (50.7%)/337 (49.3%)	238 (49.7%)/241 (50.3%) Missing 8 (2%)	109 (53.2%)/96 (46.8%) Missing 1	0.453
CEA (ng/ml)	3.56 (2.01–5.10)	2.85 (2.65–3.04)	5.64 (0.44–11.72)	0.121
CYFRA211 (ng/ml)	2.44 (2.33–2.54)	2.41 (2.29–2.52)	2.52 (2.26–2.79)	0.361
NSE (ng/ml)	15.78 (15.34–16.22)	15.76 (15.24–16.28)	15.82 (15.00–16.64)	0.914
Pathology (Squamouscell/Neuroendocrine /Adenosquamous/Small cell/Adenocarcinoma/other)	651 (95.2%)/7 (1%)/7 (1%) /7 (1%)/4 (0.6%)/8 (1.2%)	453 (95%)/4 (0.8%)/5 (1%) /7 (1.5%)/4 (0.8%)/4 (0.8%) Missing 10 (2%)	197 (95.6%)/3 (1.5%)/2 (1%) /0 (0%)/0 (0%)/4 (1.9%)	0.082
Grade (I/ I-II/ II/ II-III/ III)	32 (5%)/36 (5.6%)/240 (37.3%)/250 (38.9%) /85 (13.2%)	26 (5.7%)/26 (5.7%)/170 (37%)/178 (38.8%) /59 (12.9%) Missing 28 (6%)	6 (3.3%)/10 (5.4%)/70 (38%) /72 (39.1%)/26 (14.1%) Missing 22 (10%)	0.760
Concurrent Cancers (1:Yes/ 0:No)	49 (7.1%)/640 (92.9%)	36 (7.5%)/447 (92.5%) Missing 4	13 (6.3%)/193 (93.7%)	0.746
Maximum Diameter Of Tumor (cm)	3.06 (2.94–3.18)	3.10 (2.96–3.25)	2.98 (2.77–3.19)	0.339
Depth of tumor invasion (cm)	3.33 (3.23–3.43)	3.38 (3.28–3.45)	3.21 (3.00–3.41)	0.096
Lymph-vascular Space invasion (1:Yes/ 0:No)	134 (19.5%)/554 (80.5%)	96 (19.9%)/386 (80.1%) Missing 5	38 (18.4%)/168 (81.6%)	0.676
Neural invasion (1:Yes/ 0:No)	75 (89.1%)/612 (10.9%)	67 (13.9%)/414 (86.1%) Missing 6	18 (8.7%)/188 (91.3%)	0.051

### Predictors of different metastatic patterns in regional lymph nodes

For regional lymph nodes, univariate analysis in the training cohort was conducted. 12 significant risk factors associated with regional lymph node metastasis were identified. Only age (OR = 0.959, 95% CI 0.926–0.994; *P* = 0.021), depth of tumor invasion (OR = 1.373, 95% CI 1.091–1.727; *P* = 0.007), and lymph–vascular space invasion (OR = 3.286, 95% CI 1.829–5.526; *P* < 0.01) were identified as independent predictors of regional lymph node metastasis by Multivariable analysis of these risk factors acquired from univariate analysis (Table 2).

**Table 2:** multivariate analysis of different metastatic patterns of regional Lymph Nodes in the training cohort

	Regional lymph nodes			Continuous regional lymph nodes			Skipping regional lymph nodes		
Variables	OR	95%CI	P	OR	95%CI	P	OR	95%CI	P
Age	**0.959**	**0.926–0.994**	0.021^*^	**0.947**	**0.899–0.997**	0.037^*^	0.982	0.926–1.042	0.556
Sex	1.809	0.952–3.439	0.070	1.630	0.621–4.279	0.321	0.602	0.201–1.802	0.364
Smoke	0.732	0.391–1.371	0.330						
Alcohol	1.420	0.753–2.679	0.279	1.009	0.477–2.134	0.982	1.563	0.582–4.197	0.376
Tumor History	0.346	0.037–3.225	0.351						
Blood Type			0.329						
N/P(G)	1.256	0.790–1.997	0.336						
APTT (G)	0.681	0.424–1.093	0.112	**0.408**	**0.197–0.843**	0.016^*^			
MaximumDiameter Of Tumor (cm)	1.140	1.965–1.347	0.124	1.226	0.996–1.508	0.054	1.095	0.834–1.437	0.514
Depth of tumor invasion (cm)	**1.373**	**1.091–1.727**	0.007^*^						
Lymph-vascular Space invasion	**3.286**	**1.829–5.526**	< 0.01^*^				**3.632**	**1.499–8.797**	0.004^*^
Neural invasion	1.284	0.660–2.485	0.462	**2.658**	**1.212–5.829**	0.015^*^	0.254	0.054–1.181	0.08
length of Tumor				1.027	0.894–1.180	0.703	**1.208**	**1.019–1.431**	0.029^*^

Only variables with P value less than 0.20 in the univariate analysis were included in the multivariate analysis. Independent predictors for model were further analyzed by multivariable analysis with *P* value less than 0.05. *P < 0.05

For continuous regional lymph nodes, seven significant risk factors associated with continuous regional lymph node metastasis were revealed by univariate analysis in the training cohort. Only age (OR = 0.947, 95% CI 0.899–0.997; *P* = 0.037), APTT (G) (OR = 0.408, 95% CI 0.197–0.843; *P* = 0.016), and neural invasion (OR = 2.658, 95%CI 1.212–5.829; *P* = 0.015) were identified as independent predictors of continuous regional lymph node metastasis by multivariable analysis of these risk factors obtained from univariate analysis (Table 2).

For skipping regional lymph nodes, seven significant risk factors associated with skipping regional lymph node metastasis were revealed by univariate analysis in the training cohort. Only lymph–vascular space invasion (OR = 3.632, 95% CI 1.499–8.797; *P* = 0.004) and tumor length (OR = 1.208, 95% CI 1.019–1.431; *P* = 0.029) were identified as independent predictors of skipping regional lymph node metastasis by multivariable analysis of these risk factors obtained from univariate analysis (Table 2).

### Nomogram construction and validation

Those factors found to be independently predictive of different patterns of regional lymph node metastasis in the multivariable analyses were performed to construct the models and nomograms (Table 3).

**Table 3:** Nomogram for Prediction of different metastatic patterns of regional Lymph Nodes

Independents Variables	Model for metastatic regional lymph nodes		
	b	Odds Ratio (95% CI)	*P*-value
Age (years)	−0.042	0.959 (0.926–0.994)	0.021
Depth of tumor invasion	0.317	1.373 (1.091–1.727)	0.007
Lymph-vascular Space invasion	1.190	3.286 (1.829–5.526)	< 0.01
Independents Variables	Model for metastatic continuous regional lymph nodes		
	b	Odds Ratio (95% CI)	*P*-value
Age (years)	−0.055	0.947 (0.899–0.997)	0.037
APTT	−0.898	0.408 (0.197–0.843)	0.016
Neural invasion	0.978	2.658 (1.212–5.829)	0.015
Independents Variables	Model for metastatic skipping regional lymph nodes		
	b	Odds Ratio (95% CI)	*P*-value
Lymph-vascular Space invasion	1.290	3.632 (1.499–8.797)	0.004
length of Tumor	0.189	1.208 (1.019–1.431)	0.029

**Fig. 1:**
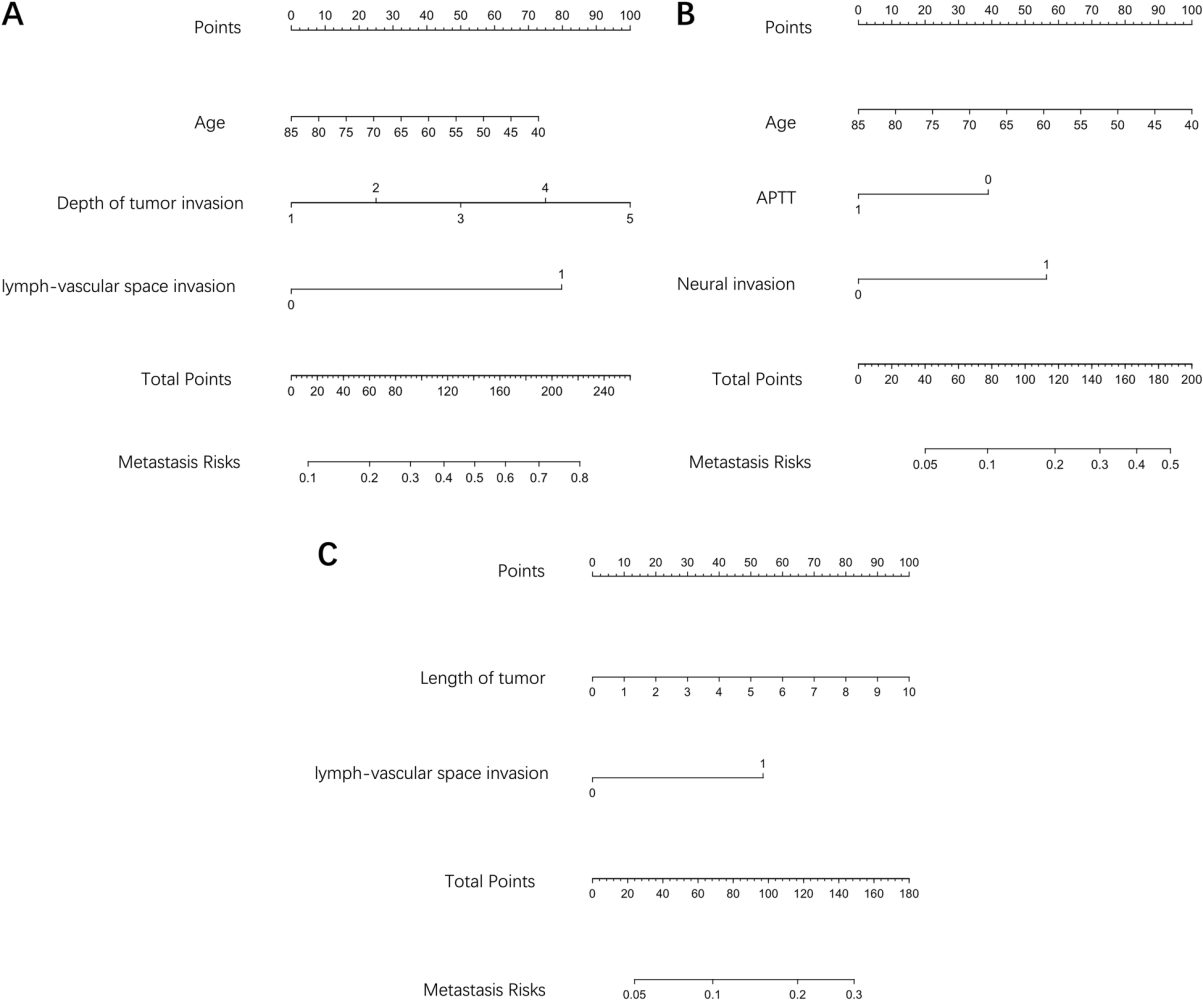
Nomograms for prediction of different metastatic patterns of regional lymph nodes in patients intraoperatively diagnosed with esophageal cancer. **a**, regional lymph nodes. Lymph-vascular space invasion: 1: positive, 0: negative. **b**, continuous regional lymph nodes. APTT: 1: > 26.6 s, 0: < 26.6 s; Neural invasion: 1: positive, 0: negative. **c**, skipping regional lymph nodes. Lymph-vascular space invasion: 1: positive, 0: negative

**Fig. 2:**
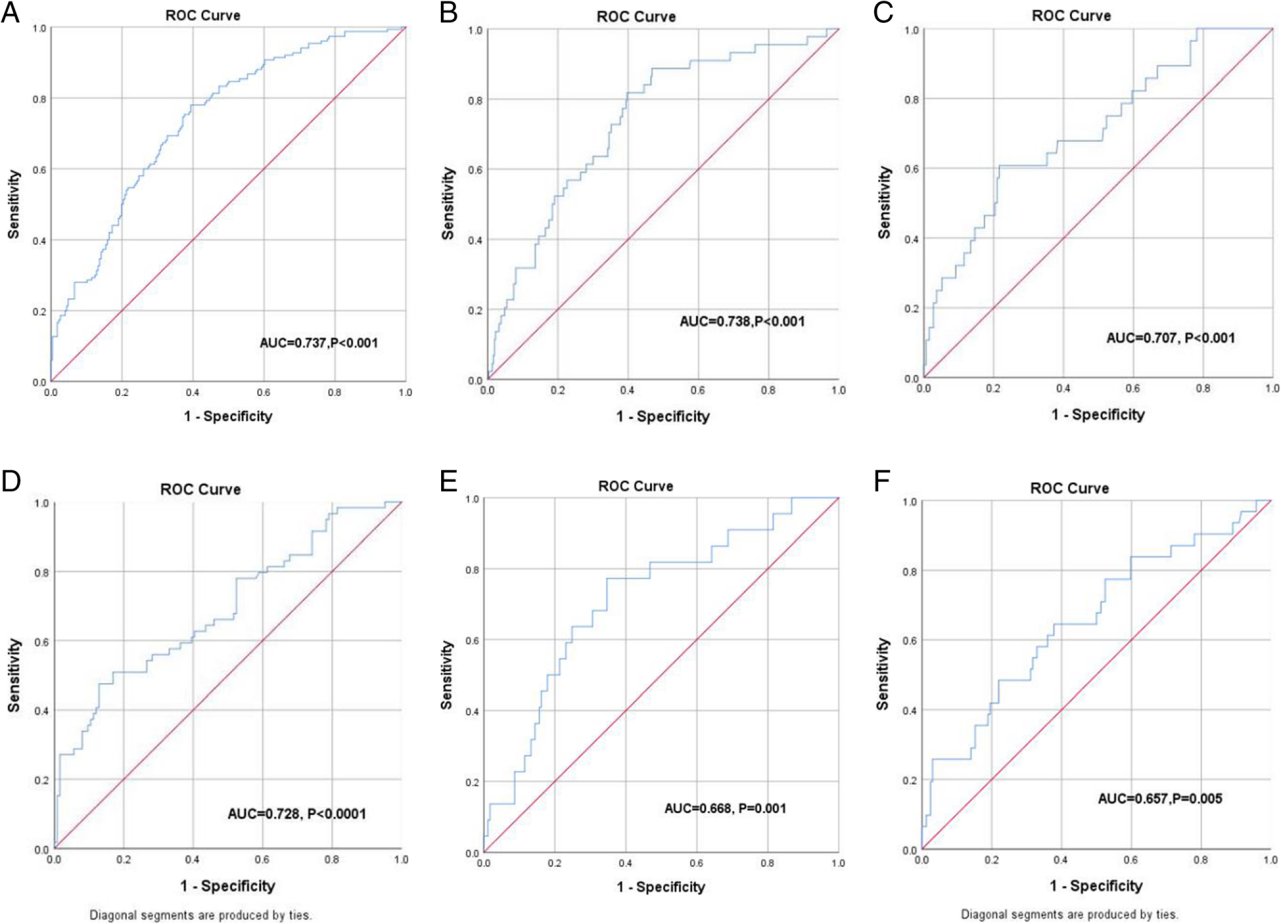
ROC curves of nomograms for prediction of different metastatic patterns of regional lymph nodes in patients intraoperatively diagnosed with esophageal cancer. **a**, regional lymph nodes. **b**, continuous regional lymph nodes. **c**, skipping regional lymph nodes in training cohort. **d**, regional lymph nodes. **e**, continuous regional lymph nodes. **f**, skipping regional lymph nodes in the validation cohort. ROC, receiver operating characteristic

With respect to the nomogram of regional lymph node metastasis (Fig. 1a), the training and validation cohort AUC values were 0.737 (*P* < 0.001) and 0.728 (*P* < 0.0001), respectively (Fig. 2a, d).

As for the nomogram for continuous regional lymph node metastasis (Fig. 1b), the training and validation cohort AUC values were 0.738 (P < 0.001) and 0.668 (*P* = 0.001), respectively (Fig. 2b, e).

In the case of the nomogram of skipping regional lymph node metastasis (Fig. 1c), the training and validation cohort AUC values were 0.707 (*P* < 0.001) and 0.657 (*P* = 0.005), respectively (Fig. 2c, f).

**Fig. 3:**
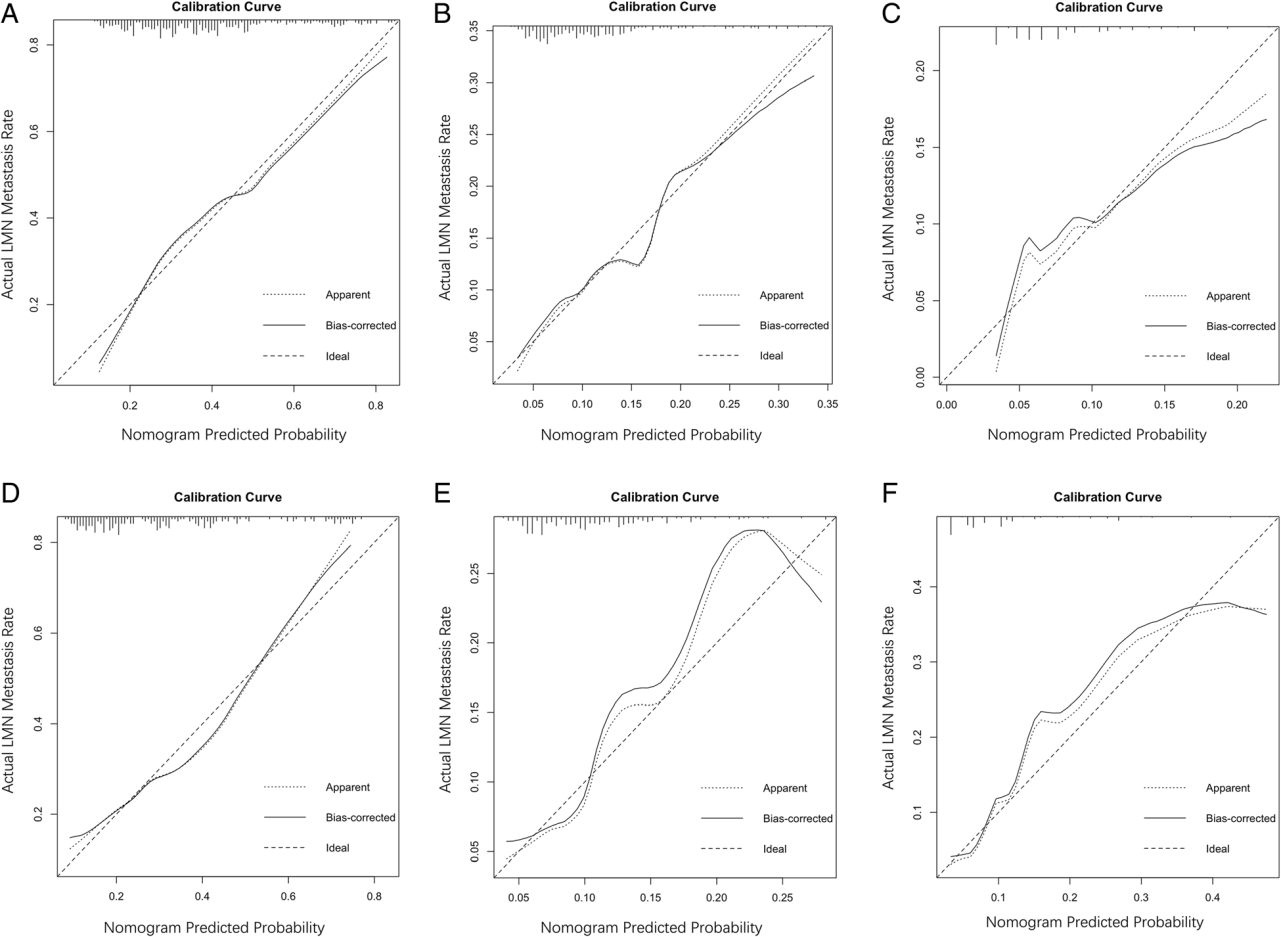
Calibration curves of nomograms for prediction of different metastatic patterns of regional lymph nodes in patients intraoperatively diagnosed with esophageal cancer. **a**, regional lymph nodes. **b**, continuous regional lymph nodes. **c**, skipping regional lymph nodes in training cohort. **d**, regional lymph nodes. **e**, continuous regional lymph nodes. F, skipping regional lymph nodes in validation cohort

Moreover, the calibration curves indicate good consistency between observed actual outcomes of lymph node metastasis and predicted values for regional lymph nodes (Fig. 3a, d), continuous regional lymph nodes (Fig. 3b, e), and skipping regional lymph nodes (Fig. 3c, f) in the two cohorts.

### Clinical application of the nomogram

**Fig. 4:**
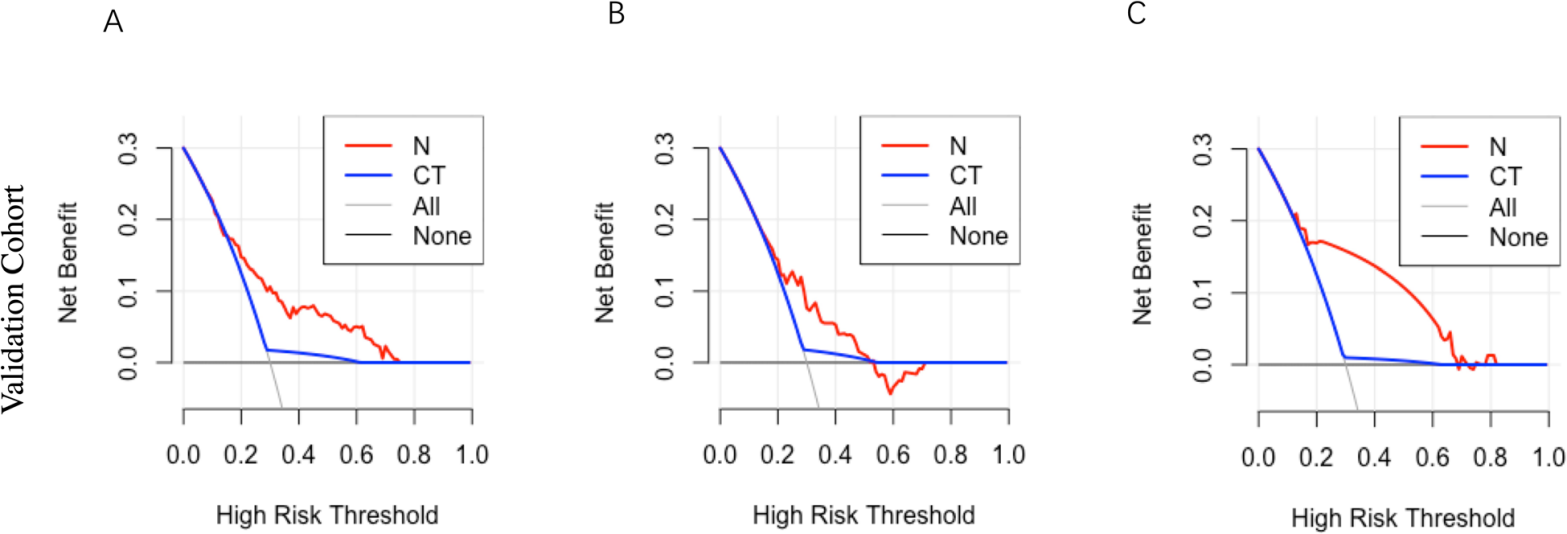
DCA for prediction of different metastatic patterns of regional lymph nodes in patients intraoperatively diagnosed with esophageal cancer. **a**, regional lymph nodes. **b**, continuous regional lymph nodes. **c**, skipping regional lymph nodes in validation cohort. DCA, decision curve analysis. N, nomogram; CT, contrast-enhanced computed tomography. The x-axis and y-axis represent threshold probability and net benefit, respectively. The black line corresponds to net benefit when all patients are considered to not have metastasis of the regional lymph nodes. The gray line corresponds to net benefit when all patients are considered to have metastasis of the regional lymph nodes

The clinical value of the nomograms was assessed by DCA based on the net benefit and threshold probabilities. As for regional lymph node metastasis, we demonstrated nomogram had superior net benefit with a wide range of threshold probabilities compared with contrast-enhanced CT in the validation cohort (Fig. 4a). For continuous regional lymph node metastasis, using the nomogram to predict lymph node metastases is more beneficial than using contrast-enhanced CT within the threshold probability range of 20 and 50% (Fig. 4b). For skipping regional lymph node metastasis, the nomogram also provided a greater superior net benefit with a wide range of threshold probabilities comparative with contrast-enhanced CT, similar to that observed with regional lymph node metastasis (Fig. 4c). DCA curves indicated that the nomograms had superior clinical usefulness compared with the routine screening method of contrast-enhanced CT to predict different patterns of regional lymph node metastasis.

## Discussion

The TNM staging system is used worldwide to determine proper treatment and establish prognosis for patients with esophageal cancer [17]. With this staging system, regional lymph nodes, “N”, are an important direct guide that enables surgeons to perform lymph node dissection. Transverse penetration of the esophageal wall and flowing longitudinally in a cephalic or caudal direction are the two main patterns of lymphatic spreading in the esophagus. The longitudinal lymphatic flow of the esophagus is more plentiful than the transverse distribution [18], so the patterns of metastatic regional lymph nodes generally include metastatic regional lymph nodes, metastatic continuous regional lymph nodes, and metastatic skipping regional lymph nodes [19, 20].

In our study, we accurately divided the thoracic region into three: the upper thoracic region; the middle thoracic region; and the lower thoracic region. Metastatic regional lymph nodes are defined as the location of positive lymph nodes (> 1) that correspond to the position of the esophageal tumor. Metastatic continuous regional lymph nodes are defined as positive lymph nodes (> 1) also found in the adjacent region besides the positive lymph nodes (> 1) in the corresponding position of the esophageal tumor. Metastatic skipping regional lymph nodes are defined as positive lymph nodes (> 1) found in the other regions by the absence of regional lymph node metastasis in the corresponding position of the esophageal tumor.

However, in clinical practice, although extended systematic nodal dissection is regarded as the best treatment for patients, the possibility of omitting positive lymph nodes also exists and a high occurrence of postoperative complications makes it difficult for surgeons to choose the best strategy of lymph node dissection [21, 22]. Surgeons face a dilemma about how to perform lymph node dissection and they must make this decision based on their experience [23, 24]. Surgeons aim to obtain additional guides for accurate lymph node dissection.

Fortunately, nomograms, which can more accurately predict metastatic lymph nodes and provide superior stratification compared with traditional methods, have been developed and validated in several types of cancer [25, 26]. In our study, a total of 693 patients were analyzed retrospectively following radical three-incision esophagectomy. Nomograms that were reasonably effective in predicting metastasis for different patterns of regional lymph nodes based on independent risk factors were constructed and validated. Our nomograms showed more accurate prediction, with AUC values of 0.737, 0.738, and 0.707 in the training cohort and 0.728, 0.668, and 0.657 in the validation cohort, respectively. Moreover, the calibration curves indicate a good consistency between the predicted outcomes and actual outcomes of lymph node metastasis. In addition, DCA demonstrated that these novel nomograms display an improved overall benefit and superior clinical utility compared with contrast-enhanced CT.

Using nomograms, the patterns of metastatic regional lymph nodes can be estimated. The possibility of metastasis of regional lymph nodes will increase when patients are younger, have deeper tumor invasion, and present with lymph–vascular space invasion. Metastasis risks of regional lymph node can be evaluated using nomograms within the range from 10 to 80% (Fig. 1a). By using ROC curve, the cut-off value is 35%. Metastasis risk of regional lymph node more than 35% is defined as high level risk. To assess the pattern of metastatic continuous regional lymph nodes, the possibility of metastasis of continuous regional lymph nodes will increase when patients are younger, have an APTT of < 26.6, and are in a condition of neural invasion. Metastasis risks of continuous regional lymph nodes can be evaluated using nomograms within the range from 5 to 50% (Fig. 1b). By using ROC curve, the cut-off value is 10%. Metastasis risks of continuous regional lymph nodes more than 10% is defined as high level. To assess the pattern of metastatic skipping regional lymph nodes, the possibility of metastasis of skipping regional lymph nodes will increase when patients have an increased tumor length and are in a condition of lymph–vascular space invasion. Metastasis risks of skipping regional lymph nodes can be evaluated using nomograms within the range from 5 to 30% (Fig. 1c). By using ROC curve, the cut-off value is 10%. Metastasis risks of skipping regional lymph nodes more than 10% is defined as high level.

Nomograms can provide surgeons with an important guide for making decisions about the strategy for lymph node dissection. If we find that the predictive metastasis risk of continuous or skipping regional lymph nodes is high, then extended systematic nodal dissection must be performed to avoid omitting positive lymph nodes. If we find that the predictive metastasis risk of continuous and skipping regional lymph nodes is low and that of regional lymph nodes is high, then we can only perform systemic regional lymph node dissection corresponding to the position of the esophageal tumor. If we find that the predictive metastasis risk of continuous and skipping regional lymph nodes is low and that of regional lymph nodes is also low, we can only perform regional lymph node sampling corresponding to the position of the esophageal tumor. By using the nomogram, we can probably decrease damage to patients and achieve the goal of high-accuracy treatment.

There are some limitations in the present study. This study was a single institution retrospective research and demonstrates the necessity for further prospective studies. It is necessary to perform further prospective studies with multicenter trials to comprehensively evaluate nomograms in the context of predicting intraoperative metastasis of different regional lymph nodes in patients diagnosed with esophageal cancer.

## Conclusions

In conclusion, powerful and simple nomograms using independent risk factors were developed and validated to predict intraoperative different patterns of regional lymph nodes in patients diagnosed with esophageal cancer. These novel nomograms suggest that they are of potential value for clinicians to select the best strategy of lymph node dissection because they displayed superior performance and discriminative power compared with traditional diagnostic systems.

## Supplementary Information

**Additional file 1.** Support file containing the gender(male:1;female:0), smoke (yes:1; no:0), alcohol (yes:1;no:0), History of esophagitis and gastritis(yes:1;no:0), diabetes(yes:1;no:0), tumor history(yes:1;no:0), tumor family history(yes:1;no:0), Bloodtype (A:0;B1;AB2;O3), Neutrophil(G), Lymphocyte(G), N/L(G), Platelet(G), N/P(G), Serum Albumin(G), ALP(G), Serum Globulin(G), Al/Gl ratio(G), APTT(G), PT(G), Pathology(Squamous cell:0,Neuroendocrine:1,Adenosquamous:2,Small cell:3,Adenocarcinoma:4,other:5), Grade(I:0,I-II:1,II:2,II-III:3,III:4), Pathological morphology(Medullary:0,Mushroom umbrella:1,Ulcerative:2,Constriction:3,early stage:4), Multifocal carcinoma(yes:1;no:0), lymph-vascular space invasion, neural invasion, skipping regional lymph nodes, Continuous regional lymph nodes, Regional lymph nodes described in categorical variables and Age, number of cigarettes, Alcohol consumption, Upper boundary of tumor(Distance to incisors), lower boundary of tumor(Distance to incisors), Length of tumor, Neutrophil, Lymphocyte, N/L, Platelet, N/P, Serum Albumin, ALP, Serum Globulin, Al/Gl ratio, APTT, PT, CEA, CYFRA211, NSE, Tumor size(cm), depth of tumor invasion(cm), upper section of esophagus(18-24 cm)middle section(24–32)lower section(32–40) described in continuous variables.

## Data Availability

All data generated or analysed during this study are included in this published article [and its supplementary information files].

## Abbreviations

LN: lymph node
CT: computed tomography
PET-CT: positron emission tomography CT
EUS: endoscopic ultrasound
TNM: tumor–node–metastasis
ORs: Odds ratios
Cis: confidence intervals
AUC: area under the curve
ROC: operating characteristics curve
DCA: Decision curve analysis
